# Effect of the Season on Blood Changes of Oxidative Stress Index in the Italian Mediterranean Buffalo (*Bubalis bubalis*)

**DOI:** 10.3390/vetsci11030116

**Published:** 2024-03-04

**Authors:** Giovanna De Matteis, Susana Flores-Villalva, Emanuela Rossi, Maria Chiara La Mantia, Roberto Steri, Vittoria Lucia Barile, David Meo Zilio

**Affiliations:** 1 Research Centre for Animal Production and Aquaculture-Consiglio per la Ricerca in Agricoltura e l’Analisi dell’Economia Agraria (CREA), 00015 Monterotondo, Italydavid.meozilio@crea.gov.it (D.M.Z.); 2 CENID Salud Animal e Inocuidad, Instituto Nacional de Investigaciones Forestales Agrícolas y Pecuarias (INIFAP), Mexico City 05110, Mexico; flores.susana@inifap.gob.mx

**Keywords:** oxidative status, antioxidants, buffalo, heat stress, vitamin D

## Abstract

**Simple Summary:**

Heat stress significantly impacts animal biological processes, including buffaloes, which exhibit signs of heat stress during hot weather. This study investigates the impact of temperature on buffaloes’ oxidative stress response over two weather seasons, identifying plasma oxidants and antioxidants. The results showed that high temperatures during the hot season caused distress, while oxidant levels increased from hot to cold season. The oxidative stress index remained unaffected by annual variation but was higher in cold seasons. This study suggests that analyzing both oxidants and antioxidants is necessary to determine the best oxidative stress index for buffalo.

**Abstract:**

Studies in cattle have shown that high temperatures increase the production of reactive oxygen species (ROS) causing an imbalance between ROS and the ability of antioxidant systems to detoxify and remove the reactive intermediates. As such studies remain limited in buffalo, the effect of temperature on oxidative stress was investigated through the oxidative stress index (OSi). Blood samples were collected from 40 buffaloes over 12 time points distributed over two years (2021, 2022). Samples were taken monthly during the hot and cold seasons. Plasma free oxygen radicals were determined using the d-ROMs test (Diacron, Italy), modified for a microplate procedure, and the results were expressed in arbitrary Carratelli Units (U.CARR). Plasma antioxidants were determined by using the BAP test (Diacron) in a dedicated spectrophotometer (Carpe Diem Free, Diacron). The OSi parameter was calculated as d-ROMs/BAP × 100. Temperature and humidity were recorded daily during the trial to calculate the Temperature Humidity Index (THI). For statistical analysis, year and season and their interactions were included in the model. The results of this study showed for the first time the effect of season on the oxidative stress in buffalo. The minimum and maximum THI values for the hot and cold season recorded during the experimental period were 79.27 ± 2.20 and 63.42 ± 3.20, respectively. Levels of d-ROMs and BAP were affected by the seasons (133.0 vs. 145.1 U.CARR, *p* = 0.0189, and 2489.19 vs. 2392.43 mml/L, *p* = 0.033, in the hot and cold season, respectively). A significant year × season interaction was found both for d-ROMs and BAP (*p* = 0.06 and *p* < 0.0001, respectively). Moreover, OSi was affected by season, showing a growing trend from hot to cold season (5.35 vs. 6.17, *p* < 0.0001), but, interestingly, it was unaffected by annual variation. Therefore, Osi could be considered a better and independent marker of oxidative status in buffalo, with respect to the evaluation of single determinations of d-ROMs and BAP. Lastly, there were no differences in the plasma 25OHD levels between seasons; concentrations were 12.24 and 10.26 ng/mL in the hot and cold season, respectively.

## 1. Introduction

Livestock productivity can be affected by a variety of environmental factors. High temperatures and humidity can negatively impact the production, reproduction, and health of livestock animals. Current scenarios predict that global warming will continue to increase in the next years and is very likely to exceed 1.5 °C [1]. This will cause very wet and very dry weather, intensifying climate events and changes in extremes during seasons [1,2]. Climate change negatively impacts water availability and the quantity and quality of feed, all crucial elements for livestock production. The increase in temperature and precipitation variation also triggers fluctuations in animal disease epidemiology and affects the animal’s growth and milk production [3,4].

All animals have a range of temperatures that are ideal for both physiological processes and maximizing their productive potential. This range is known as the thermal comfort zone or thermal neutral zone [4]. When the environmental temperatures increase above this range (>25 °C), animals expend energy to dissipate the heat and maintain their body temperature, entering a state of heat stress [5,6]. Heat stress causes physiological and behavioral changes in animals, such as an increase in respiratory rate, heart rate, and rectal temperature. The higher heart rate allows for increased blood flow to the body surface and, therefore, the dissipation of more heat. Similarly, stressed animals will reduce their feed intake and drink more water, which will allow them to regulate their internal metabolic heat production [7]. The mechanisms by which heat is dissipated vary between species and life stages [5,7,8]. Cattle, for example, can release heat through cutaneous evaporation because they have more sweat glands than buffalo. Cattle will therefore seek shade when they are under heat stress [9]. Conversely, buffalo have comparatively few sweat glands, meaning that the animal’s ability to cool itself through evaporative sweating is diminished. Buffalos therefore wallow in mud or water to keep their body temperature stable in hot weather [6,7].

The water buffalo (*Bubalus bubalis*) is an important livestock species present mainly in Asia, South America, and the Mediterranean basin [10]. Buffalos are an important source of milk, meat, and draught power. Indeed, the second-largest source of milk in the world comes from buffalo [11]. In many countries, buffalo cheese is highly prized and desirable; some examples are Italy’s *Mozzarella*, Iraq’s *Gaymer*, Egypt’s salty *Domiati* cheeses, and Bulgaria’s yogurt [10,12]. Buffalo meat is an important source of proteins for the human requirements and its production is growing in different countries due also to the high-quality characteristics that affect positively human health, compared to the red meat coming from other species [13].

Therefore, understanding how environment, and in particular, how hot and cold weather affects buffaloes is essential for ensuring their welfare and productive parameters.

Heat stress is assessed through physiological variables such as the rectal temperature (RT), respiratory rate (RR), heart rate (HR), skin temperature (ST), and body temperature (BT). However, heat stress also causes cellular and molecular responses, such as an imbalance in the production of oxidants and antioxidants, resulting in oxidative stress [14]. Heat stress has been reported to cause oxidative stress in livestock animals, including dairy cattle [15], sheep [16], pigs [17], and buffaloes [18]. Oxidative stress has been linked to several pathologies in humans, such as cardiovascular diseases, metabolic syndrome, neurodegenerative disorders, autoimmune diseases, and cancer [19]. Less is known about the effects of oxidative stress on animal health. Recent research indicates that oxidative stress has a significant role in inflammatory and immunological dysregulation in dairy cows during the transition period, which raises the risk of reproductive diseases like acidosis, metritis, mastitis, and placental retention [20,21]. In addition, oxidative stress due to heat stress has been linked to low-quality meat with a reduced shelf life [22].

Direct measurement of reactive oxygen species (ROS) is challenging because of their short lifetime and high reactivity; therefore, their activity is measured indirectly by evaluating their chemical interaction with other molecules such as lipids, proteins, or DNA [23]. The derivatives of reactive oxygen metabolites (d-ROMs) test and the biological antioxidant potential (BAP) test are simple, reliable, and accurate methods for the measurement of oxidants and antioxidant levels in plasma and other biological fluids [24]. The ratio between the levels of d-ROM and BAP, known as the oxidative stress index (OSi), has been used as a biomarker of oxidative stress in humans [25], dairy cattle [26], sheep [27] and horses [28] providing evidence of their use as indicators of health or disease in animals. However, OSi reference values for buffaloes have not been reported. Furthermore, despite the fact that some studies have reported the levels of oxidants and antioxidants in buffaloes, reference values for these biomarkers have not yet been defined [18,29,30].

Thus, in the present study, we analyzed the effect of temperature on the oxidative stress response in buffaloes during the hot and cold seasons across two years; the concentration of d-ROMs, BAP, and OSi values was investigated. Furthermore, we analyzed the vitamin D circulating levels and their correlation with the oxidative stress response.

## 2. Materials and Methods

### 2.1. Animals and Housing

The present study was conducted at the experimental farm of the Research Centre for Animal Production and Aquaculture of CREA in Monterotondo, Rome (42°08′ N, 12°06′ E, 165 m above sea level) for two years (2021 and 2022), in cold and hot periods for our latitude (February to April and June to September, respectively). The experiment has started after the first cold wave (T < 0 °C) has occurred, that happened for both years between the end of January and February. A total of forty pluriparous Italian Mediterranean buffaloes were selected for this study. The animals were between 4 and 9 years old, had 2 to 6 calvings, and had on average 105.77 ± 73.58 DIM. The average body weight of the selected animals was 734.25 ± 55.44 kg. The average milk yield was 2454.78 ± 592.41 kg in 298 ± 53 days, and with 7.79 ± 0.56% and 4.72 ± 0.21% of fat and protein, respectively. All buffaloes were housed in an open paddock and were fed ad libitum once a day (8:00 a.m.) on a total mixed ration (TMR) based on sorghum silage (49% as is), alfalfa hay (19.5% as is), soya bean meal (6.1% as is), maize meal (11% as is) and barley meal (11% as is), buffer, mineral, and vitamin supplementation containing 0.90 UFL/Kg of dry matter (DM), and 15% crude protein on (DM). Buffaloes were milked twice a day (8 a.m. and 6 p.m.). The milking parlor was 10 + 10 herringbone stalls, equipped with lactometers for individual measurements (DeLaval) and Dellpro 5.5 management software.

Blood samples were collected for the two years in cold and hot seasons. In total, 12 time points, approximately 40 days apart, were taken over the two-year period (2021 and 2022).

### 2.2. Measurement of Physiological Variables

Body condition score (BCS) and rectal temperature (RT) for each animal was recorded during each sampling (12 in total). BCS was measured by one trained person, with a 9-point BCS scale modified for buffalo [19] and expressed as a mean value. In addition, body lateral and upper images were individually recorded for subsequent validations of BCS, as obtained by the image analysis. RT was measured after two hours of exposure to direct sunlight using an animal clinical thermometer and was taken by keeping the thermometer in the rectum for 2 min.

### 2.3. Enviromental Conditions

Information on the daily ambient temperature (AT) and relative humidity (RH) was recorded daily during the trial through a meteorological station (Vantage Pro2™ Plus-SKU 6327, Davis Instruments) near the barn. Temperature and humidity data were used to calculate the Temperature Humidity Index (THI). The formula used was THI = (1.8 × T − ((1 − Ur/100) × (T − 14.3)) + 32), with T = temperature (°C) and Ur = relative humidity (%) [31].

### 2.4. Analysis of Oxidants and Antioxidants

Blood samples were taken from the jugular vein in 10 mL lithium heparin tubes. Plasma was immediately separated after centrifugation (1200× *g* for 15 min at 5 °C) and stored at −20 °C until assayed. The reactive oxygen metabolites (ROMs) were quantified with the standardized d-ROMs test (Diacron International, Grosseto, Italy) using the microplate procedure. The results are expressed in arbitrary ‘Carratelli Units’ (U.CARR), where 1 U.CARR is equivalent to the oxidizing power of 0.08 mg H_2_O_2_/dL. The biological antioxidant potential (BAP) was determined using the BAP test (Diacron International, Grosseto, Italy) in a dedicated spectrophotometer (Carpe Diem Free, Diacron International, Grosseto, Italy) with the results recorded in μmol/L. The Oxidative Stress index (OSi) was calculated as d-ROMs/BAP × 100.

### 2.5. Analysis of Circulating Levels of Vitamin D

The plasma samples were analyzed for concentration of total 25OHD using an ELISA assay (Vit D ELISA, EUROIMMUN, Lübeck, Germany) and carried out following the manufacturer’s instructions. Since no buffalo standards for 25OHD were available, buffalo samples were validated by checking the 25OHD concentrations by LC-MS/MS. This validation was performed at the Vitamin D Laboratory (VitDAL) at the University of Edinburgh.

### 2.6. Statistical Analysis

The data were analyzed in SAS 9.4 (SAS/STAT, Version 9.4. SAS Institute, Cary, North Carolina, NC, USA). A preliminary analysis was conducted for all detected factors, eliminating those found to be not significant. The final model used was the following:

(d-ROMs, BAP, OSi) = season + year + season × year

Data were expressed as the mean ± SD for all parameters. The graphs of these parameters were conducted using GRAPHPAD PRISM 8 computer software (San Diego, CA, USA). The Pearson correlation analysis was conducted on R (version 4.3.2) using R studio (version 2023.12.1). This analysis was performed using the tidyverse, Hmisc, and corrplot packages.

## 3. Results

### 3.1. Temperatures Recorded in the Hot and Cold Weather Seasons in 2021 and 2022

**Figure 1:**
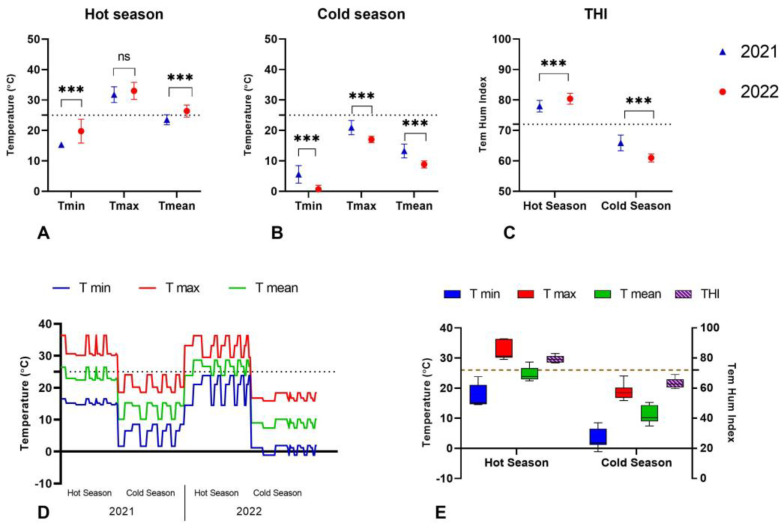
Temperatures (T) and temperature humidity index (THI) recorded during the hot and cold weather seasons in 2021 and 2022. (**A**) Scatter plot of T min, T max, T mean registered during the hot weather seasons per year. (**B**) Scatter plot of T min, T max, T mean registered during the cold weather seasons per year. (**C**) Scatter plot of THI registered during the hot and cold weather seasons per year. (**D**) Scatter plot of T min, T max, T mean registered across the hot and cold seasons in 2021 and 2022. The dotted line shows the optimal temperature (25 °C) threshold. (**E**) Boxplot of combined (including both years) T min, T max, T mean, and THI recorded for the hot and cold season. The dotted line shows the optimal THI (<72) threshold for buffaloes according to [9]. ns means not significant. *** *p* < 0.0001.

The temperatures recorded during the hot and cold weather seasons of 2021 and 2022 were very similar (Figure 1D). However, the hot season in 2022 was warmer than the hot season in 2021, with a mean temperature of 26.39 ± 1.98 °C in 2022 versus 23.54 ± 1.62 °C in 2021 (*p* < 0.0001) (Figure 1A). There was no difference in the maximum temperatures recorded during the hot season; however, the minimal temperature was significantly lower during 2021 (Figure 1A).

Similarly, the cold weather season from 2022 had higher freezing temperatures, with a mean temperature of 8.84 ± 1.13 °C, in comparison to the mean temperature of 13.26 ± 2.25 °C observed during the cold season from 2021 (*p* < 0.0001) (Figure 1B). During 2022, significantly lower temperatures were recorded, with a minimal temperature of 0.66 ± 1.29 °C and a maximal temperature of 17.02 ± 1.04 °C (*p* < 0.0001) (Figure 1B).

The differences in temperature were observed as well as the mean THI (Temperature Humidity Index), which was higher during the hot weather season in 2022 (80.40 ± 1.80), and lower during 2021 (77.98 ± 1.86) (*p* < 0.0001). The cold weather THI were 65.88 ± 2.58 and 60.97 ± 1.32 during 2021 and 2022, respectively (Figure 1C).

Collectively (including both years), the maximum and minimum temperatures registered in the hot weather season were 32.44 ± 2.74 °C and 17.70 ± 3.66 °C, respectively. The mean temperature for the hot season was 25.07 ± 2.31 °C (Figure 1E). For the cold weather season, the maximum temperature was 18.98 ± 2.66 °C, with a minimal temperature of 3.11 ± 3.31 °C. The mean temperature for the cold season was 11.05 ± 2.84 °C. The THI for the hot and cold weather seasons were 79.27 ± 2.20 and 63.42 ± 3.20, respectively (Figure 1E).

### 3.2. Physiological Variables of Buffaloes during Hot and Cold Weather

**Figure 2:**
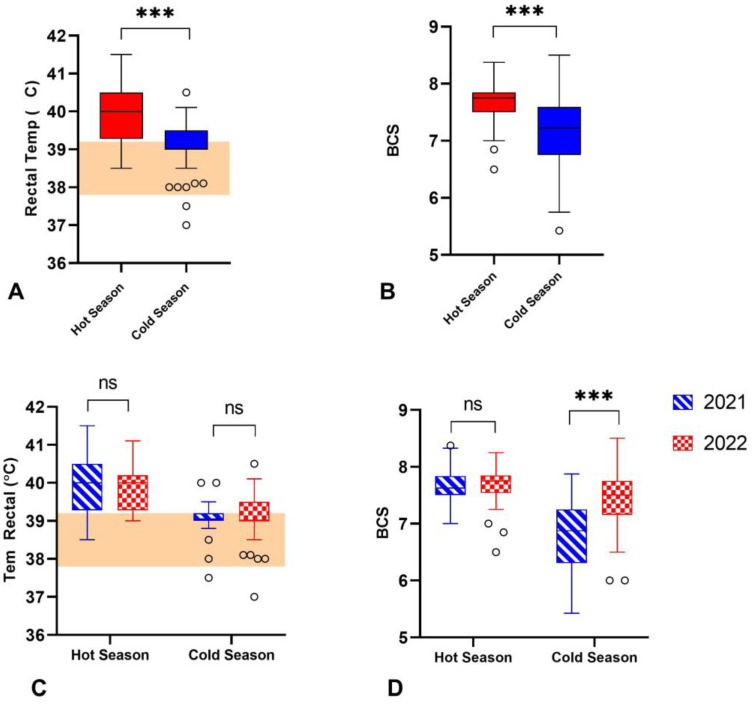
Body Condition Score (BCS) and rectal temperature (RT) from buffaloes during the hot and cold weather seasons in 2021 and 2022. (**A**,**B**) Boxplot of the combined (including both years) RT and BCS from buffaloes during the hot and cold seasons. (**C**,**D**) Boxplot of RT and BCS registered during the hot and cold weather seasons per year. The pink area shows references values for buffalo heifers according to [32]. Dots represent outlier values. ns means not significant. *** *p* < 0.0001.

The BCS and RT were significantly higher during the hot season compared to the cold season (Figure 2). The mean BCS during the hot season was 7.6, in contrast with a mean 7.1 BCS recorded during the cold season (*p* < 0.0001) (Figure 2B). There was an increase in the RT of buffalos during the hot season, with a mean RT of 39.9 °C, in contrast to the mean RT of 39.1 °C during the cold season (*p* < 0.0001) (Figure 2A). Interestingly, our results showed that the mean RT values for buffaloes were higher compared to the values observed for buffalo heifers [32].

### 3.3. Effect of Heat Stress on the Oxidative Stress Response

**Figure 3:**
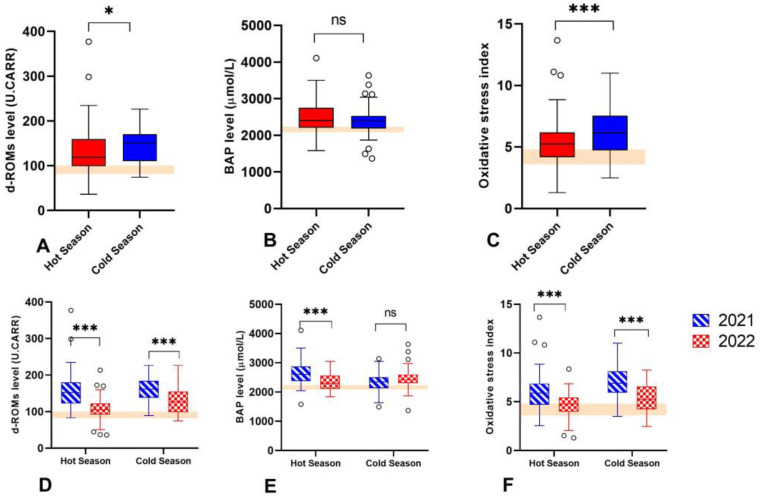
Oxidative stress response in buffaloes during hot and cold weather seasons. (**A**–**C**) Boxplot of combined levels (including both years) of d-ROMs, BAP and Osi during the hot and cold weather seasons. (**D**–**F**) Boxplot of levels of d-ROMs, BAP and Osi during the hot and cold weather seasons in 2021 and 2022. The pink area shows references values for buffalo according to [33]. Dots represent outlier values. ns means not significant. * *p* < 0.05, *** *p* < 0.0001.

To investigate the effect of heat stress on the oxidative status, the concentrations of d-ROMs and BAP in plasma were evaluated, and the OSi was determined. A two-way ANOVA was performed to analyze the effect of season and year on the oxidative status. The analysis showed that for each analyte, there was not a statistically significant interaction between the effects of season and year. However, season and year have significant individual effects on the d-ROMs, BAP, and OSi levels (Figure 3D–F). The combined analysis (including both years) showed that the d-ROMs (145.1 ± 3.36 and 131.2 ± 4.77 U.CARR in cold and hot weather, respectively) and OSi (6.157 ± 0.16 and 5.302 ± 0.17 in cold and hot weather, respectively) values were higher in cold weather than in hot weather. Whereas the BAP (2392 ± 30.46 and 2477 ± 37.21 μmol/L, in cold and hot weather, respectively) levels showed no differences between the seasons (Figure 3A–C).

### 3.4. Vitamin D (25OHD) Levels Observed during the Hot and Cold Weather Seasons

**Figure 4:**
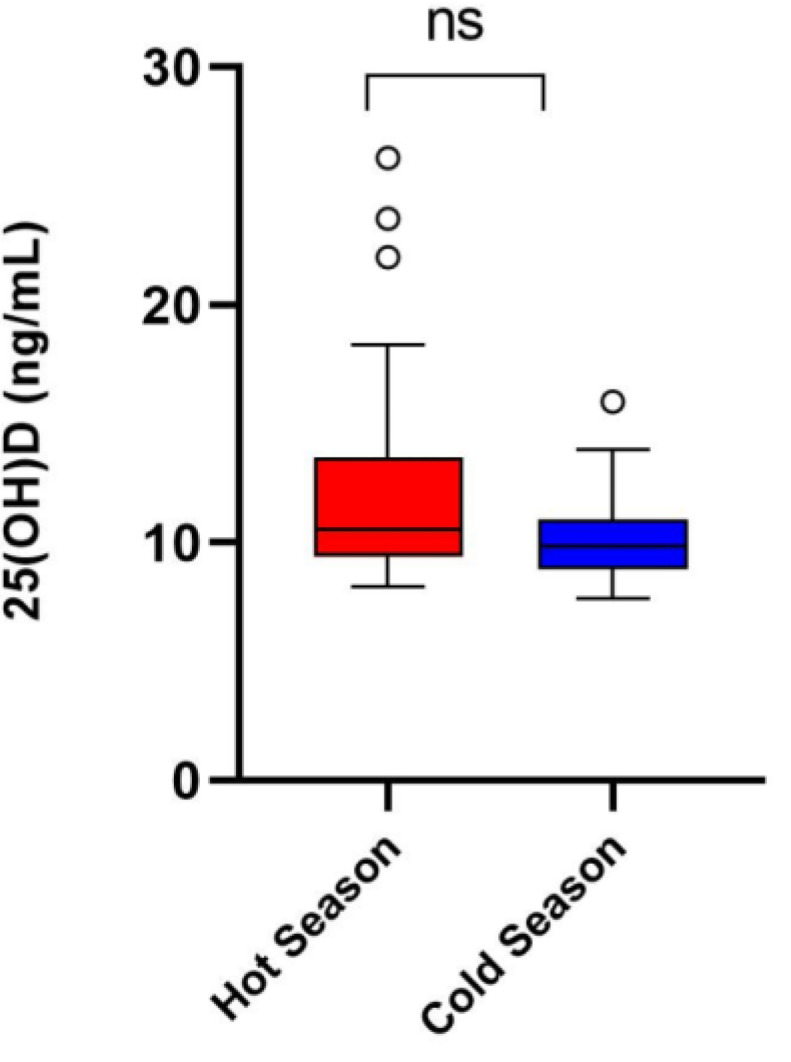
Boxplot of circulating concentration of vitamin D (25OHD) in plasma from buffaloes during the hot and cold weather seasons. Dots represent outlier values. ns means not significant.

We examined the circulating vitamin D levels of buffaloes across two seasons. In general, we observed very low levels of 25OHD during the hot and cold weather seasons; the mean 25OHD concentration was slightly higher in the hot season compared to the cold season (12.24 ± 0.77 vs. 10.26 ± 0.38 ng/mL, respectively), although no significant differences were found (*p* = 0.0795) (Figure 4).

### 3.5. Correlation Analysis for All the Variables

**Figure 5:**
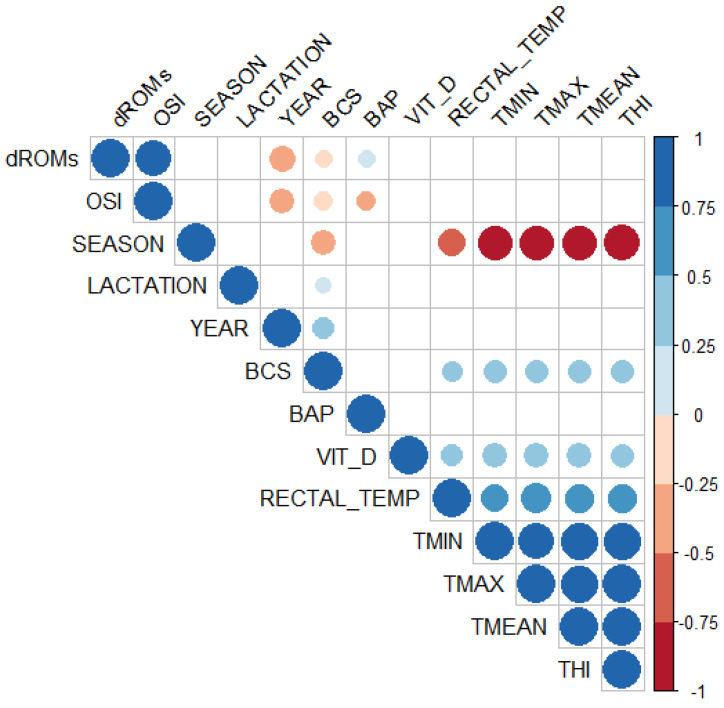
Graphical display of the correlation matrix analysis. In the plot, positive correlations are displayed in blue and negative correlations in red. Color intensity and the size of the circle are proportional to the correlation coefficients. On the right side of the plot, the legend color shows the correlation coefficients and the corresponding colors. Only significant (*p* ≤ 0.01) correlations are shown. Insignificant correlations are left blank.

Finally, we performed a correlation analysis to determine the relationships between the variables (Figure 5). As expected, the T min, T max, and T mean showed strong positive associations with THI (r > 0.95, *p* < 0.0001). In addition, a moderate positive correlation was observed between THI and RT (r = 0.58, *p* < 0.0001). There was a positive correlation between vit D and RT (r = 0.33, *p* < 0.001), vit D and temperatures T min, T max, and T mean (r = 0.40, *p* < 0.0001), and between vit D and THI (r = 0.39, *p* = 0.001). The results showed that there was a weak positive correlation between BCS and year, RT, T min, T max, T mean, and THI.

Additionally, the analysis showed strong negative correlations between the weather, season, and the temperatures T min, T max, T mean (r = −0.84, *p* < 0.0001), as well as a moderate negative correlation between season and RT (r = −0.53, *p* < 0.0001). There was a moderate negative correlation between season and BCS (r = −0.42, *p* < 0.0001), between OSi and year (r = −0.42, *p* < 0.0001), and between d-ROMs and year (r = −0.47, *p* < 0.0001).

## 4. Discussion

Buffaloes thrive in hot and humid weather; however, their black and hairless skin absorbs a large amount of solar radiation. Thus, they will display distress when working in the sun during hot weather [34,35]. Buffaloes are best suited to environmental temperatures between 13 and 18 °C, in combination with relative humidity around 55–65%. Unfortunately, extreme weather changes and high temperatures are becoming more common [1]. Thus, understanding how weather changes affect buffaloes is essential for ensuring their welfare and productive parameters.

In our study, the temperatures recorded during the hot and cold seasons were very similar in both years, although comparatively, 2022 was hotter than 2021. Accordingly, during the hot season, the THI was above 78, regarded as severe stress for buffaloes [9]. An animal under heat stress will immediately respond by changing its body temperature. Our results showed that there was an increase of almost 1 °C in the RT of buffaloes during the hot season in comparison to the cold season. The increase in the RT was associated with the high THI, suggesting that animals were distressed because of the high temperatures [36]. Although we did not observe a reduction in the BCS in our study, it has been shown that a rise of 1 °C or less in RT is enough to reduce intake and production in dairy cows and buffaloes [29,37]. RT is a sensitive indicator of the physiological response to heat stress, as it is nearly constant under normal conditions [37]. However, some authors have reported no significant differences in the RT at different THI levels or seasons on non-lactating buffaloes [38,39]. This is not surprising, since calves and heifers generate less metabolic heat than lactating cows [40]. Our results are in agreement with previous studies showing an increase in RT in animals suffering from heat stress [29,36,41].

Heat stress leads to oxidative stress, which can damage cellular macromolecules, in particular DNA, proteins, and lipids [19,21]. Interestingly, our results showed a growing trend in the plasma d-ROMs and OSi values from the hot to the cold season, whereas plasma BAP remained at similar levels between seasons. Although d-ROMs and BAP levels were affected by season, the OSi value was unaffected by annual variation, suggesting that OSi is a better and independent marker of the oxidative status in buffalo, with respect to the evaluation of single determinations of d-ROMs and BAP. However, none of the oxidative stress markers were associated with RT or THI. Analysis of the oxidative stress markers in ruminants is limited. Studies have mostly focused on the effects of diseases like mastitis or acidosis in cattle [20,26,42,43], but few studies have been reported in buffalo [30,38,39]. There are a number of biomarkers that can be used to monitor oxidative stress; however, differences in the endpoints and methodologies used to investigate the oxidant and antioxidant markers make the comparison between studies meaningless, even for studies quite similar [38]. For instance, Megahed G.A., et al. [34]. reported that lipid peroxide (LPO) and nitric oxide (NO) levels were significantly elevated during the summer season in comparison to winter in healthy buffaloes. In addition, decreased activity of the superoxide dismutase (SOD) enzyme was observed in animals suffering heat stress [34]. Similarly, buffalos exposed to a THI > 80 during summer in China had higher malondialdehyde (MDA) content and lower levels of plasma antioxidant enzymes (GPx, SOD, and CAT) as compared to other seasons [39]. The above revealed a reduced total antioxidant capacity to manage the excessive load of ROS produced during the hot season. This is in contrast to our findings, which showed that the oxidative status of buffaloes is influenced by cold temperatures rather than heat. One explanation for these discrepancies is the methodology used. Each assay has its advantages and disadvantages [43]; however, some assays have been criticized for low specificity and artifact formation, such as MDA assays, whose results can differ according to the assay conditions used [44]. The d-ROMs and BAP tests are fast, simple, and sensitive assays that have been widely validated [43]. To the authors knowledge, no data about the d-ROMs, BAP, and OSi levels on buffalo under different weather conditions has been reported. In addition, the reference values for these biomarkers have not yet been defined. Recently, a preliminary study reported the physiological range of d-ROMs and BAP levels during the different phases of the estrus cycle in buffaloes [33]. The study was performed on 30 healthy buffaloes, of which ten were in estrus, ten in diestrus, and ten in anestrus. The authors found the highest d-ROMs value and the lowest BAP level during the estrus phase, though the d-ROMs and BAP levels reported by the authors were lower than those observed in our study [33]. This finding may be related to the difference in the number of animals analyzed between the studies. Our d-ROMs and BAP values are in accordance with a recent study where the effects of spirulina on the oxidative status were assessed [45]. Contrary to this, Tudisco et al. [46] reported d-ROMs and BAP levels completely opposite to those described for buffaloes and cattle [26,33,47]. Therefore, it is important to establish the physiological ranges for these biomarkers according to standard published procedures so they can reliably reflect the oxidative status of the individual animal [48].

Finally, we measured the circulating levels of vitamin D, an essential micronutrient required for optimal antioxidant defense and functional capability in some leukocyte populations [49]. We did not observe differences in the 25OHD plasma levels between seasons, and concentrations were below 30 ng/mL of 25OHD, a threshold reported to be optimal for cattle [50]. The very low levels of circulating 25OHD could be the result of the buffaloes’ skin structure, which has a higher concentration of melanin in the skin in comparison to cows [3]. Melanin reduces the ultraviolet radiation available for vitamin D synthesis in the skin, and perhaps the low circulating levels of vitamin D are related to the melanin content of the buffalos’ skin [51]. To the authors knowledge, this is the first time a study reports the 25OHD plasma levels for buffaloes. Therefore, further research is needed to determine the reference values for circulating 25OHD levels in buffaloes and reassess their vitamin D requirements.

## 5. Conclusions

Our study reports for the first time, the concentration of d-ROMs, BAP, and OSi values for buffaloes in relation to changes in temperature. Despite animals being distressed during the hot season, the OSi values indicated that cold temperatures during the winter affected the oxidative status of buffaloes. Therefore, understanding the adaptive responses of buffaloes to extreme weather, including heat and cold, is warranted. The identification of biological markers of distress can pave the way for the incorporation of mitigation strategies. Moreover, our results showed that apparently buffaloes had lower circulating levels of vitamin D in comparison to bovines; however, the implications for animal health require further investigation. Future research should focus on the establishment of a reference panel of biomarkers that reliably reflect the oxidative status of buffaloes, which could be used to adopt the best strategy to improve welfare and productivity in this species.

## Data Availability

The datasets analyzed during the current study are available from the corresponding author on reasonable request.
